# The intention of North-Western Ethiopian dairy farmers to control mastitis

**DOI:** 10.1371/journal.pone.0182727

**Published:** 2017-08-07

**Authors:** Sefinew Alemu Mekonnen, Gerrit Koop, Theo J. G. M. Lam, Henk Hogeveen

**Affiliations:** 1 Department of Farm Animal Health, Faculty of Veterinary Medicine, Utrecht University, Utrecht, The Netherlands; 2 Faculty of Veterinary Medicine, University of Gondar, Gondar, Ethiopia; 3 GD Animal Health, Deventer, the Netherlands; 4 Business Economics Group, Wageningen University, Wageningen, The Netherlands; Institute of Animal Sciences, GERMANY

## Abstract

Understanding the intentions of dairy farmers towards mastitis control is important to design effective udder health control programs. We used the Theory of Planned Behavior (TPB) to explore the intentions of North-Western Ethiopian dairy farmers towards implementing non-specified mastitis control measures (nsMCMs) and towards implementing 4 specific MCMs. Face to face interviews were held with 134 dairy farmers to study associations between their intentions and any of three factors (attitude, subjective norm and perceived behavioral control) that, according to the TPB, determine intentions. The majority of the farmers (93%) had a positive intention to implement nsMCMs, whereas a smaller majority of farmers had the intention to implement the specific MCMs to improve udder cleaning (87%), to improve stall hygiene (78%), to improve feeding of cows (76%), and to perform foremilk stripping (74%). Farmers had a more positive attitude, but lower subjective norm and lower perceived behavioural control towards implementing nsMCMs compared with implementing most specific MCMs, although the subjective norms for stall hygiene and perceived behavioural control for improving feeding of cows were also low. Attitude was positively associated with intentions to implement nsMCMs, to improve cleaning of the udders, to improve stall hygiene and to implement foremilk stripping. Both the intention to improve udder cleaning and to implement foremilk stripping, were positively associated to subjective norms towards these MCMs. Our data can help tailor intervention programs aiming to increase the intention of Ethiopian dairy farmers to implement MCMs and thus to improve udder health in this country. We show that such programs should primarily focus on changing attitude and secondarily on improving the farmers’ subjective norms.

## Introduction

Recently, a remarkable increase in milk production is seen in African countries like Egypt, Ethiopia, Uganda and Namibia [1]. Market infrastructure and increasing populations in combination with urbanization have led to the intensification of dairy farming around urban areas [1,2].

There are three dairy production systems in Ethiopia; urban, peri-urban and rural. The urban and peri-urban dairy production is market-oriented and include smallholder and specialized commercial dairy farms mainly concentrated in and around Addis and other regional towns [3]. These urban and peri-urban dairy productions keep Holstein-Friesian and Zebu cross-breed cows and are expanding and serving as the major milk supplier to the fast growing urban market [2,4,5]. The rural dairy production system predominantly uses indigenous breeds with a low milk yield. This rural dairy production system is part of the subsistence farming system and includes pastoralist, agro pastoralist and mixed crop-livestock producers [6]. Unlike the urban and peri-urban dairy production, the rural dairy production system is not market-oriented and most of the milk produced is used for home consumption. Currently, this dairy production system is being substituted by the urban and peri-urban dairy production systems.

Mastitis is the most prevalent production disease in dairy herds worldwide [7]. It is a well-documented disease with a heavy burden in both, developed and developing countries [8,9]. In Ethiopia, mastitis is one of the most frequent [10] diseases of dairy cows reported with high prevalence [11–13]. Recently, we showed that a higher Holstein-Friesian blood level is associated with more mastitis [14]. This is important given the growing numbers of urban and peri-urban dairy farmers that use such cross-breeds, aiming for higher milk yields. In Holstein-Friesian and Zebu cross-breed dairy herds, mastitis was one of the two major clinically manifested health problems [15] and the second cause of culling [16] reported to be an economically important problem [17]. Associated with loss in milk yield due to subclinical mastitis alone, Mungube et al. [18] reported a financial loss of US$38 and Tesfaye et al. [19] of US$79 per cow per lactation. Several other diseases such as internal parasites, lumpy skin disease, heartwater, blackleg, hypocalcaemia and trypanosomosis are also prevalent in Ethiopia, but their importance largely differs between regions and with cattle breeds [14,20–23]. In the Holstein-Friesian and Zebu cross-breed cows in the urban and peri-urban dairy farms in the North-West of Ethiopia, mastitis likely is one of the most important diseases.

Improving udder health requires the application of appropriate mastitis control measures (MCMs) by those involved in managing dairy herds and the milking process [24]. Success of implementation of udder health programs depends on the willingness of farmers to change their behavior [25]. Strategies that enhance farmers’ motivation to improve udder health in their herds might, therefore, be an important part of effective mastitis control programs. In large dairy herds in the developed world, farmers have been shown to be motivated to improve mastitis control based on their perception of the economic losses of mastitis [26,27]. Money, however, is not the only factor that motivates farmers in their decisions to improve mastitis management [26]. Non-monetary factors, such as farmer characteristics, and psychological factors such as attitude (AT), subjective norms (SN) and perceived behavioral control (PBC) also the motivation of farmers to improve mastitis and to implement MCMs [25,28].

Understanding the drivers of dairy farmers’ motivation is, therefore, essential for implementing effective intervention strategies [29,30]. However, only a limited number of studies have investigated factors motivating farmers to improve animal disease control in Ethiopia or other sub Saharan countries [31,32], none of them focusing on mastitis. The theory of planned behavior (TPB) framework has been used in several studies to obtain insight in the psychological factors that influence intentions related to animal health. This approach has, for instance, also been used by Lind et al. [33] to investigate farmers’ participation in herd health programs and their behavior concerning treatment of mild clinical mastitis and by Bruijnis et al. [30] to gain insight on dairy farmers’ intention to improve dairy cow foot health.

The present study was conducted with the goal to identify what determines the motivation of North-Western Ethiopian dairy farmers towards controlling mastitis, and specifically to study how their AT, SN and PBC towards MCMs are related to their intention to implement these MCMs. A second goal was to identify socio-demographic characteristics potentially affecting farmers’ intentions trough associations with their AT, SN and PBC.

## Materials and methods

### Theoretical framework

**Fig 1:**
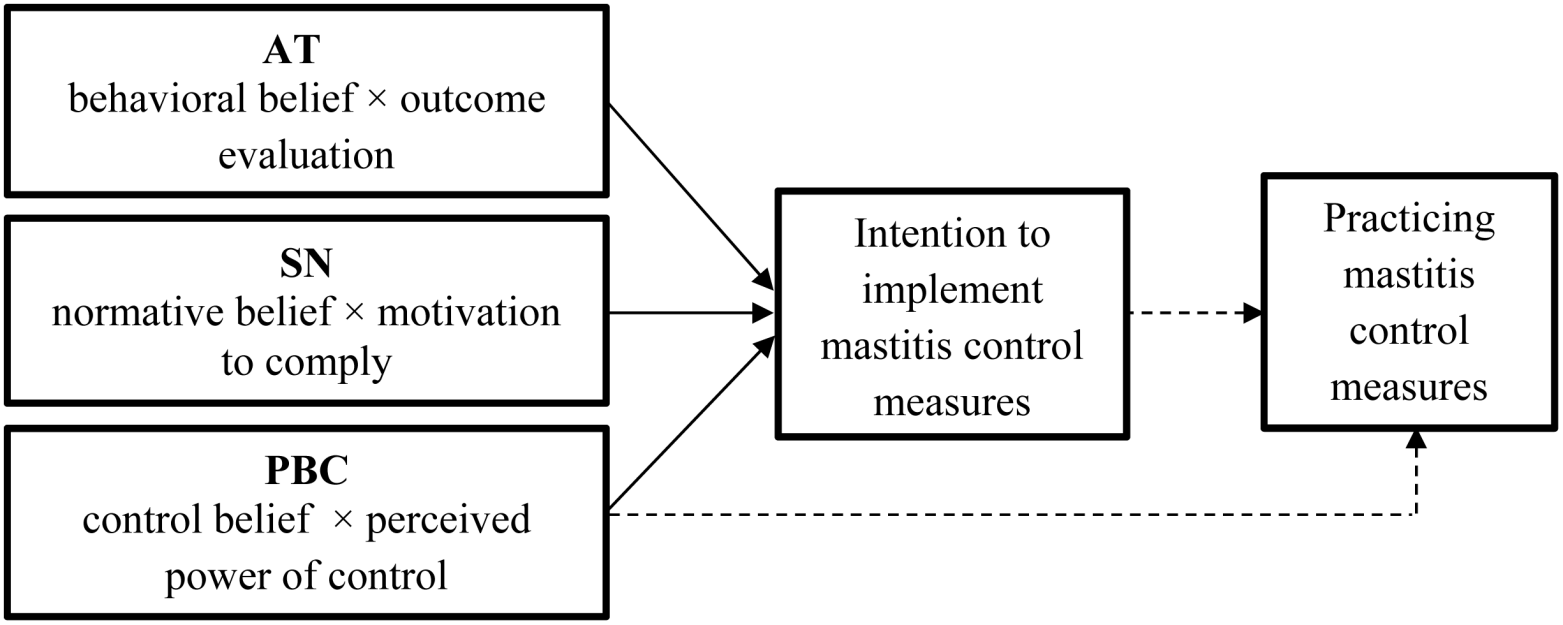
Framework of the theory of planned behavior model as applied in the performed analyses on the intention to participate in mastitis control measures (adapted from Ajzen [35]). AT = Attitude, SN = Subjective norm, PBC = Perceived behavioral control. Dotted lines indicate associations that are not studied in this paper.

According to the TPB, a behavioral intention indexes a person’s motivation to perform (or not perform) a behavior and is the immediate determinant of an action [34]. The theory also postulates that a person’s intention to perform (or not perform) behavior of different kinds can be predicted with high accuracy from his AT, SN, and PBC (Fig 1) [35]. By changing these three ‘predictors’, it is possible to increase the person’s intention to implement a desired action and thus to increase the chance that a person will actually execute that behavior [34,36]. The variables in the TPB model are psychological (internal) constructs that can be seen as latent variables which can be approximated by interviewing [36].

With regard to mastitis control, the AT is determined by the farmer’s beliefs about the consequences of performing MCMs (behavioral beliefs) and the corresponding positive or negative judgements the farmer gives to this effect (outcome evaluation). Subjective norms are determined by beliefs about how other people, who may be in some way important to the dairy farmer, would like him to behave (normative beliefs) and the motivation to comply with these referents (motivation to comply). Perceived behavioral control is determined by the dairy farmer’s belief whether he has the necessary resources (control belief) including knowledge, money, time and labor and how confident he feels about being able to perform or not perform the MCM (perceived power of control) [35,36].

### Questionnaire design

The questionnaire design was tailored to MCM under Ethiopian circumstances and on data requirements of the TPB model. The first step in constructing the questionnaire was identifying potential MCMs for the Ethiopian peri-urban dairy farming situation. One statement was aimed at obtaining the intentions towards MCMs in general, that we refer to in this paper as non-specified MCMs (nsMCMs). Four statements were designed about intentions towards specific MCMs (Table 1).

**Table 1:** Statements used in the face to face interviews to quantify farmers’ intentions towards implementing non-specified mastitis control measures and 4 specific mastitis control measures, using a 7 points bipolar Likert scale (-3 to 3).

Intention statements
In the near future I plan to implement one or more MCM(s) to reduce mastitis in my farm
In the near future I plan to improve the cleaning of the udders to reduce mastitis in my farm
In the near future I plan to improve stall hygiene to reduce mastitis in my farm
In the near future I plan to improve feeding of my cows to reduce mastitis in my farm
In the near future I plan to foremilk strip to reduce mastitis in my farm

MCM(s) = mastitis control measure(s).

Behavioral, normative, and control beliefs that can affect dairy farmers’ behavior and corresponding evaluations were identified based on our experience. These latent variables were measured by asking respondents about statements concerning their beliefs and the corresponding evaluations.

The behavioral beliefs of AT (bbAT) towards the intention to implement nsMCMs was measured by 4 items, all referring to the impact of mastitis: treatment costs, milk production losses, risk of culling, and impact on milk quality. Behavioral beliefs of attitudes towards the specific MCMs were measured by asking dairy farmers about the importance of the specific MCMs when it would be implemented on their farm in the near future. To measure the bbAT towards improving udder cleaning, the opinion on the effect of udder cleaning on their farms was studied by asking about the effect of cleaning on milking hygiene, spread of infection and diagnosis of clinical mastitis. To measure the bbAT towards improving stall hygiene, the opinion on the effect of stall hygiene on udder health in general, spread of infection and exposure of teats to infection was quantified. The bbAT towards improving feeding of cows was studied by asking about the expected effect on the cows’ nutritional balance and the cows’ susceptibility for mastitis. To measure the bbAT towards implementing foremilk stripping, the farmer’s opinion on the effect of foremilk stripping on diagnosis of mastitis and on the possibility to give treatment early was asked. Corresponding outcome evaluations of the behavioral beliefs of both the specific and the nsMCMs were measured by statements asking the farmer to give his own positive or negative judgements about the MCMs.

To measure the SNs, we considered veterinarians, artificial inseminators, milk customers, neighbors and other dairy farmers most important to the dairy farmer to approve or disapprove MCMs. Normative beliefs were evaluated by statements asking the farmer about the opinion of these 5 groups of people to approve or disapprove MCMs. Motivations to comply were measured by statements asking the farmer whether the opinion of those people influences his intention to implement MCMs.

Perceived behavioral control was evaluated in relation to the ability of the farmer, and to time and money needed to implement MCMs. Perceived behavioral control beliefs were measured by statements evaluating whether MCMs were difficult to implement, were time consuming and were considered expensive by the farmer. The corresponding perceived power of control was measured by statements evaluating how confident a farmer feels about being able to implement, having time to implement and having money to cover the costs to implement a MCM.

Participants gave their level of agreement for each statement on a 7 point Likert’s scale. Statements referring to beliefs were evaluated using a unipolar Likert scale (1 to 7). Statements referring to outcome evaluations, motivation to comply and perceived power of control questions were evaluated using a bipolar 7 point Likert scale (-3 to 3). An illustration of the questionnaire structure is given for udder cleaning in Table 2. The English version of the whole questionnaire is available in supporting information file (S1 Table).

**Table 2:** Statements used to measure behavioral, normative and control beliefs and outcome evaluation, motivation to comply and perceived power of control for udder cleaning on 134 North-Western Ethiopian dairy farmers.

	Latent variables	Statements	Measurement
Attitude	Behavioral belief	What is your opinion about udder cleaning?	
		1. Udder cleaning reduces mastitis	Strongly disagree 1 2 3 4 5 6 7 Strongly agree
		2. Cleaning the udder minimizes spread of bacteria causing mastitis	Strongly disagree 1 2 3 4 5 6 7 Strongly agree
		3. Clean udders facilitate diagnosis of clinical mastitis	Strongly disagree 1 2 3 4 5 6 7 Strongly agree
	Outcome evaluation	What is your opinion about the importance of udder cleaning?	
		1. Milking hygiene by cleaning the udder is important to reduce mastitis	Strongly disagree -3–2–1 0 1 2 3 Strongly agree
		2. Minimizing spread of mastitis causing bacteria by cleaning the udder is important to reduce mastitis	Strongly disagree -3–2–1 0 1 2 3 Strongly agree
		3. Facilitating diagnosis of mastitis by cleaning the udder is important to reduce mastitis	Strongly disagree -3–2–1 0 1 2 3 Strongly agree
Subjective norm	Normative belief	What, according to your knowledge, is the opinion of the following people?	
		1. Veterinarians think that udder cleaning is…	Very unimportant 1 2 3 4 5 6 7 Very important
		2. Artificial inseminators think that udder cleaning is…	Very unimportant 1 2 3 4 5 6 7 Very important
		3. Milk customers think that udder cleaning is…	Very unimportant 1 2 3 4 5 6 7 Very important
		4. My family thinks that udder cleaning is…	Very unimportant 1 2 3 4 5 6 7 Very important
		5. Other dairy farmers think that udder cleaning is…	Very unimportant 1 2 3 4 5 6 7 Very important
	Motivation to comply	Does the opinion of the following people influence your intention to improve udder cleaning?	
		1. Veterinarians	Not at all -3–2–1 0 1 2 3 Very much
		2. Artificial inseminators	Not at all -3–2–1 0 1 2 3 Very much
		3. Milk customers	Not at all -3–2–1 0 1 2 3 Very much
		4. Neighbors	Not at all -3–2–1 0 1 2 3 Very much
		5. Other dairy farmers	Not at all -3–2–1 0 1 2 3 Very much
Perceived behavioral control	Control belief	What is your opinion on the implementation of udder cleaning?	
		1. Udder cleaning is difficult	Strongly disagree 1 2 3 4 5 6 7 Strongly agree
		2. Cleaning of the udder is time consuming	Strongly disagree 1 2 3 4 5 6 7 Strongly agree
		3. Cleaning the udder is expensive	Strongly disagree 1 2 3 4 5 6 7 Strongly agree
	Perceived power of control	What is your capability to implement udder cleaning?	
		1. I know how to clean the udder	Strongly disagree -3–2–1 0 1 2 3 Strongly agree
		2. I have time to clean the udder	Strongly disagree -3–2–1 0 1 2 3 Strongly agree
		3. I can afford to cover costs of cleaning the udder	Strongly disagree -3–2–1 0 1 2 3 Strongly agree

Information that provides the basis for dairy farmers’ beliefs about the consequences of implementing MCMs, about the normative expectations of important people, and about the obstacles that may prevent them from performing a behavior can affect behavioral, normative, and control beliefs towards the intention of performing MCMs. Similarly, different groups of people can have experiences and information that differ in important ways from the experiences of others [34,36]. Therefore, additional data were collected about social and informational background factors expected to influence behavioral, normative, or control beliefs of Ethiopian dairy farmers. Open-ended or closed questions were used to collect information on these factors.

The questionnaire was prepared in English, and translated into the farmers’ language (Amharic). The questionnaire was then translated back to English by an external translator, to validate the translation. The final questionnaire was tested by pilot interviews with five farmers to check whether the farmers had any difficulty in answering the questions. The farmers used for the pilot interviews were selected based on convenience. Questions which were difficult to understand by farmers were modified.

### Data collection

The data was collected from selected dairy farmers in two regions in North-Western Ethiopia: Gondar and Bahir Dar. These two sites are areas where there are a large number of market-oriented dairy farmers who kept Holstein Friesian × Zebu breed dairy cows. We made an extensive list of dairy farms at the sites before selecting our respondents. Artificial insemination records from private and government artificial insemination centers, lists of dairy farmers from two dairy associations, and records from government veterinary clinics were used to prepare the list. Finally, 1,209 dairy farms in Gondar and 272 in Bahir Dar were listed. Then, a random number was given to each of the farms. Data collection started based on the order of the random numbers, separately for the Gondar and the Bahir Dar list of farms. The list for Gondar was expected to contain many records of farms that did not exist anymore or that could not be included in the study for other reasons. For that reason a much longer list was used. We are not aware of previous work related to Ethiopian or any other sub-Saharan countries dairy farmers’ intentions to control mastitis. Therefore, the required sample size could not be determined based on work done previously. However, in a multiple regression approach with a response rate of 50%, a sample size of 80 is acceptable for TPB studies [36]. With this in mind, we had a sampling scheme based on the time and logistics available, with the goal to include a representative sample from the population of dairy farmers. In total, 369 farms (321 from Gondar and 48 from Bahir Dar) were visited. As expected the number of farms in Gondar that could be used in the study was much smaller than the number of farms on the list; 178 dairy farms had indigenous breeds only, 30 had stopped dairy farming, of 16 farms the address could not be found and 6 farms were not willing to be interviewed (230 farms in total). When a farmer was not willing to be interviewed, had quit dairy farming or the address could not be found, that farm was skipped and the farm with the subsequent number on the list was approached. For these reasons, 230 farms in Gondar and 5 farms in Bahir Dar were not included, leaving 91 farms from Gondar and 43 from Bahir Dar to be included in the study.

For the questionnaire-based study described here, no formal approval by the Institutional Review Board of the University of Gondar or Utrecht University was required. Participating farmers were instructed about the content and goal of the questionnaire before the start of the interview (informed consent). All data were analyzed and reported anonymously. The data was collected by the first author and two final year students in Veterinary Medicine who were specifically trained for this purpose.

### Statistical analysis

The data was inspected for data entry errors by checking whether all responses were within the range of the response format. The score given for each of the behavioral, normative and control belief statements was multiplied by the corresponding outcome evaluation, motivation to comply, or perceived power of control, respectively, to create a new variable that represented the product score. In this way, product composites were created for the three TPB factors (AT, SN and PBC).

Cronbach’s alpha was calculated among the product composites of AT, of SN and of PBC to test internal consistency. The product composites were considered to have internal consistency and, therefore, to measure similar construct, if Cronbach’s alpha was >0.7 [37]. In that case, product composites were averaged to obtain one single measure (mean score) for AT, SN and PBC (Eqs 1–3). For those constructs where the Cronbach’s alpha was <0.7, product composites were used as separate TPB factors.

$$\text{AT}=\frac{\sum_{i=1}^{n}\left(bb_{i}*oe_{i}\right)}{n}$$ (1)

Where i = the product composite item i and n = number of items for AT, bb = behavioral belief, oe = outcome evaluation.

$$\text{SN}=\frac{\sum_{i=1}^{n}\left(nb_{i}*mc_{i}\right)}{n}$$ (2)

Where i = the product composite item i and n = number of important referents for SN, nb = normative belief, mc = motivation to comply.

$$\text{PBC}=\frac{\sum_{i=1}^{n}\left(cb_{i}*ppc_{i}\right)}{n}$$ (3)

Were i = the product composite item i and n = number of relevant control belief items, cb = control belief, ppc = perceived power of control.

We did not find an established rule in literature to categorize TPB factors into levels. However, a number of factors influence the beliefs people hold such as ethnicity, socio-economic status, nationality, religious affiliation, general attitudes and values [34]. Based on these reasons, it seems impossible to have standardized categorization criteria. Therefore, the TPB factors were classified into three categories based on the distribution of the product composites. Weak AT, weak SN and weak PBC (product composite ≤ 0), moderate AT, moderate SN and moderate PBC (>0 to <19), and strongly positive AT, strongly positive SN and strongly positive PBC (≥19). When the category weak or the category strong had <10 observations, categories were merged, resulting in two categories (weak and moderate or moderate and strong). For AT, SN and PBC of nsMCMs, two categories were created, below and above the median. A dependent variable was constructed for the intention towards each of the MCMs by classifying dairy farmers into low intenders (Likert score for intention ≤0) versus high intenders (Likert score for intention >0). The categorized intention variables were used as dependent variable in logistic regression models (one model for each intention) with AT, SN and PBC as predictors and, as is commonly done in TBC studies, the full models were presented. First, we did univariable analysis with region (Bahir Dar and Gondar) as fixed effects and intentions as dependent variable. The analysis didn’t show any association between region and intentions. Therefore, we evaluated associations for Gondar and Bahir Dar in the same model.

To investigate the effect of background factors on TPB variables, all the background factors were used as binary variables in logistic regression models with TPB factors (AT, SN and PBC) as dependent variables. Variables significant at P<0.15 by Chi-squared test, or Fisher’s exact test, when the number of observations in a cell was <5, were used in multivariable models. Variables which were significant at P<0.10 were retained in the final models using a backward stepwise procedure. The statistical analyses were performed using SPSS version 22.0 (IBM SPSS for Windows, Armonk, NY: IBM Corp).

## Results

### Descriptive statistics

**Fig 2:**
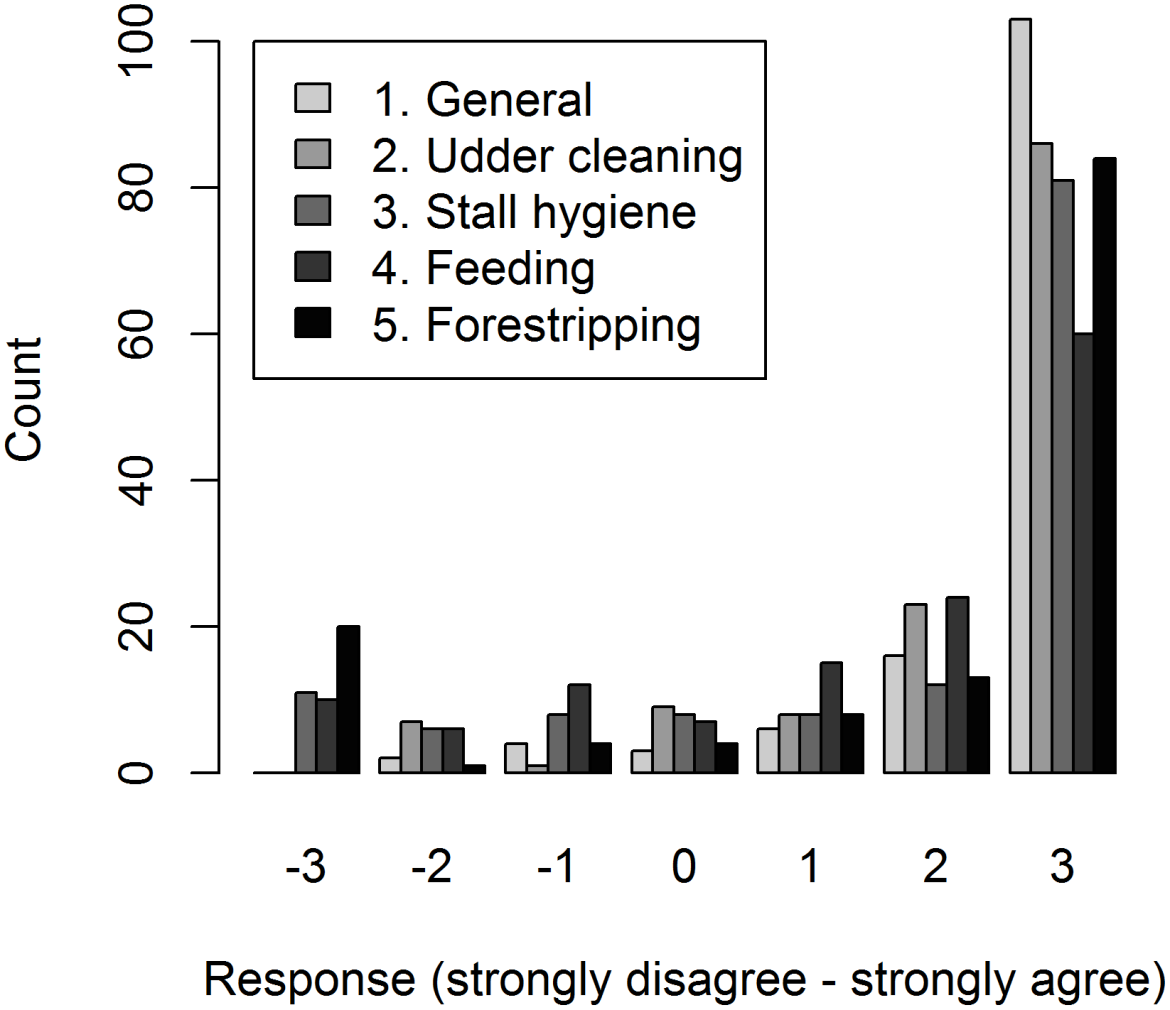
Dairy farmers’ intentions to implement mastitis control measures based on 134 farmers in North-Western Ethiopia.

On average, dairy farmers that participated in the study had 15 years of dairy farming experience, 5 animals in the herd and were 50 years of age. The majority of dairy farmers had a positive intention to implement nsMCMs. More farmers (93%) intended to implement nsMCMs than any of the specific MCMs. A large number of dairy farmers had a positive intention to improve cleaning of the udder, while the lowest proportion of farmers had a positive intention to improve feeding of cows (Fig 2).

Table 3 shows the descriptive statistics of the TPB factors. Overall, the farmers often had a positive AT and positive SN, except for nsMCMs and improving stall hygiene, where respectively, 50% and 64% of farmers had a positive SN. With respect to PBC, farmers scored very high for 3 out of 5 MCMs, but the percentage of farmers positive for PBC was very low for nsMCMs and for improving feeding of cows compared to the percentage of farmers positive for PBC in other MCMs.

**Table 3:** Descriptive statistics of attitude (AT), subjective norms (SN) and perceived behavioral control (PBC) measured with respect to the MCM intentions in 134 farmers in North-Western Ethiopia.

Mastitis control Measure	Variable	Cronbach’s alpha	Weak (n)	Moderate (n)	Strong positive (n)	Positive responses (n (%))
**Non-specified mastitis control measures**	AT (bb)	0.91	0	43	91	134 (100)
	SN (nb*mc)	0.71		67	67	67 (50%)
	PBC_1_ (cb*ppc)	0.57		70	64	64 (48)
	PBC_2_ (cb*ppc)			76	58	58 (43)
	PBC_3_ (cb*ppc)			74	60	60 (45)
**Improving cleaning of the udders**	AT (bb*oe)	0.90	35	67	32	99 (74)
	SN (nb*mc)	0.80	26	98	10	108 (81)
	PBC (cb*ppc)	0.79	34	69	31	100 (75)
**Improving stall hygiene**	AT (bb*oe)	0.72	39		95	95 (71)
	SN_1-3_ (nb*mc)	0.77	19		115	115 (86)
	SN_4_ (nb*mc)		74		60	60 (45)
	SN_5_ (nb*mc)		52		82	82 (61)
	PBC (cb*ppc)	0.80	124		10	131 (98)
**Improving feeding of cows**	AT (bb*oe)	0.87	20	99	15	114 (85)
	SN (nb*mc)	0.78	36		98	98 (73)
	PBC (cb*ppc)	0.70	65		69	69 (51)
**Foremilk stripping**	AT (bb*oe)	0.80	21	40	73	113 (84)
	SN (nb*mc)	0.87	33	90	11	101 (75)
	PBC (cb*ppc)	0.78	11	109	14	123 (92)

AT = Attitude, bb = behavioral beliefs, oe = outcome evaluation, SN = subjective norm, nb = normative belief, mc = motivation to comply, PBC = perceived behavioral control, cb = control belief, ppc = perceived power of control, 1–3 = the 1^st^, the 2^nd^ and the 3rd items that had Cronbach’s alpha value >0.7 and averaged for SN of stall hygiene, but for non-specified mastitis control measures, it was used as separate variable without averaging them, 4–5 = the 4^th^ and 5^th^ SN items used as separate variables for tall hygiene.

The number of farmers grouped on the levels of social and informational background factors is presented in Table 4.

**Table 4:** Social and informational background factors expected to influence behavioral, normative and control beliefs of dairy farmers in Noth-Western Ethiopia.

Group	Background factor	Groups (n)	
**Social**	Gender	Male (107)	Female (27)
	Age	≤ 50 years (77)	>50 years (57)
	Level of education	≤ 8 grade (65)	>8 grade (69)
	Membership to dairy association	No (64)	Yes (70)
	Herd size	≤ 3 cows (63)	>3 cows (71)
**Informational**	Experience of dairy farming	≤15 years (73)	>15 years (61)
	Knowledge whether mastitis exists	No (8)	Yes (126)
	Training about dairy cows management	No (98)	Yes (36)
	Experience of mastitis in own farm last year	No (65)	Yes (69)

### TPB factors associated with intentions to control mastitis

The behavioral belief statements of the intention for nsMCMs were highly correlated to the corresponding outcome evaluations, suggesting that they address the same construct. Therefore, only the scores given for behavioral belief were used in this analysis. Dairy farmers’ ATs were significantly and positively associated with the intention to implement nsMCMs and specific MCMs: to improve cleaning of the udders, to improve stall hygiene and to implement foremilk stripping. Subjective norms were significantly associated with the intention to improve udder cleaning and implement foremilk stripping (Table 5). PBC was not significantly associated to any of the intentions.

**Table 5:** Logistic regression model results describing the association of dairy farmers’ attitude, subjective norms and perceived behavioral control with intention towards implementing non-specified mastitis control measures, improving udder cleaning, improving stall hygiene, improving feeding of cows and implementing foremilk stripping, based on interviews with 134 farmers in North-Western Ethiopia. Significant odds ratios (P<0.05) are given in bold.

Mastitis control intention	Variable	Weak	Moderate OR^a^ (95%CI^b^)	Strong positive OR (95%CI)
**Non-specified mastitis control measures**	AT (bb)		Ref^c^.	**18.6 (3.3–103.1)**
	SN (nb*mc)		Ref.	4.5(0.5–43.4)
	PBC_1_ (cb*ppc)		Ref.	2.0 (0.3–12.3)
	PBC_2_ (cb*ppc)		Ref.	0.3 (0.1–2.1)
	PBC_3_ (cb*ppc)		Ref.	1.2 (0.2–6.2)
**Improving cleaning of the udders**	AT (bb*oe)	Ref.	**5.0 (1.0–25.5)**	6.6 (0.8–56.4)
	SN (nb*mc)	Ref.	**4.5 (1.2–16.2)**	4.6 (0.4–54.4)
	PBC (cb*ppc)	Ref.	1.1 (0.2–5.7)	1.1 (0.2–8.1)
**Improving stall hygiene**	AT (bb*oe)	Ref.		**2.5 (1.1–5.9)**
	SN_1-3_ (nb*mc)	Ref.		2.9 (0.9–9.9)
	SN_4_ (nb*mc)	Ref.		0.6 (0.2–1.5)
	SN_5_ (nb*mc)	Ref.		0.7 (0.3–2.0)
	PBC (cb*ppc)	Ref.		3.3 (0.4–28.5)
**Improving feeding of cows**	AT (bb*oe)	Ref.	1.6 (0.5–4.5)	2.1 (0.4–10.5)
	SN (nb*mc)	Ref.		1.0 (0.4–2.5)
	PBC(cb*ppc)	Ref		1.0 (0.4–2.2)
**Foremilk stripping**	AT (bb*oe)	Ref.	**4.0 (1.2–13.4)**	**8.0 (2.7–30.4)**
	SN (nb*mc)	Ref.	**4.9 (1.8–13.3)**	4.6 (0.5–47.4)
	PBC (cb*ppc)	Ref.	2.3 (0.5–9.9)	1.7 (0.2–14.5)

^a^ Odds ratio,

^b^ 95% confidence interval,

^c^ Reference category,

AT = Attitude, bb = behavioral beliefs, oe = outcome evaluation, SN = subjective norm, nb = normative belief, mc = motivation to comply, PBC = perceived behavioral control, cb = control belief, ppc = perceived power of control, 1–3 = the 1^st^, the 2^nd^ and the 3rd items that had Cronbach’s alpha value >0.7 and averaged for SN of stall hygiene, but for non-specified mastitis control measures, it was used as separate variable without averaging them, 4–5 = the 4^th^ and 5^th^ SN items used as separate variables for tall hygiene.

### Background factors

Of the nine background factors evaluate, two were associated with SN and two other factors were associated with AT. The background factors associated at P<0.10 with TPB factors of the intentions to control mastitis in the multivariable analyses are presented in Table 6. Perceived behavioral control had no association with any of the background factors. All the data in this paper are available in supporting information file (S2 Table).

**Table 6:** Social and informational background factors significantly associated (P<0.10) with dairy farmers’ (n = 134) attitude and subjective norms that were significantly associated with intentions to implement mastitis control measures based on 134 farmers in North-Western Ethiopia.

Mastitis control measure	Background factors	TPB factors	level	OR^a^ (95% CI^b^)
**Non-specified mastitis control measures**	Experience with mastitis during the last year	AT^d^	No	Ref.^c^
			Yes	2.43 (1.07–5.53)
**Improving cleaning of the udder**	Gender	SN^e^	Female	Ref.
			Male	0.28 (0.08–0.98)
**Foremilk stripping**	Dairy farming experience	AT	≤15 years	Ref.
			>15 years	2.37 (1.01–5.56)
	Education level	SN	≤8 grades	Ref.
			>8 grades	0.44 (0.22–0.87)

^a^ Odds ratio,

^b^ 95% confidence interval,

^c^ Reference category,

^d^Attitude,

^e^Subjective norm.

## Discussion

This study was conducted to obtain insight in the determinants of the intentions of North-Western Ethiopian dairy farmers towards controlling mastitis and to identify socio-demographic characteristics associated with the farmers’ AT, SN and PBC in controlling mastitis. The study was done based on the theory of planned behavior (TPB) framework to explore intentions towards nsMCMs and 4 specific MCMs. According to the TPB, behavior can be predicted at high accuracy by using behavioral intention as a proxy measure of an action [34]. The theory assumes rational behavior and the intention of a person to perform a certain behavior is assumed to be influenced by their AT, SN and PBC [35]. If we take udder cleaning as an example, the actual cleaning of the udder is a behavior whereas intention is readiness to clean the udder [38]. Attitude refers to whether a farmer is in favor of udder cleaning, SN is dairy farmer’s perception of social pressure to clean the udder and PBC represents the sense of self-efficacy or ability to clean the udder.

### Intentions of dairy farmers to control mastitis

In this study, intentions towards nsMCMs and towards 4 specific MCMs were compared, which, to our knowledge, is a novel approach. We found that the majority of farmers had the intention to implement the suggested MCMs, but interestingly, the intention to practice nsMCMs was higher than the intentions to implement the specific MCMs. Apparently, farmers have a strong intention to do “something”, which is quite an intangible measure, but the intention decreases when the measures become more tangible. The reason for this could be a perceived lack of evidence of effectiveness of the specific MCMs as well as a drawback due to a lack of ease of implementation, because of the time it takes, and costs [39]. However, as the question on nsMCMs essentially comprises all 4 specific MCMs, only farmers who are not intending to perform any of the 4 specific MCMs should disagree with the statement about intentions towards nsMCMs. Therefore, it may reflect that farmers had multiple MCMs in mind when answering the generic question. Because we asked the nsMCMs questions first, we believe that the farmers were not biased by the specific MCMs that we mentioned later on, so the question on nsMCMs truly reflects the high intention of the farmers to do something about mastitis on their farm. By then asking about more specific MCMs, we could identify that farmers intend to work on udder cleaning, stall hygiene and foremilk stripping, but much less to improve feeding, possibly because many of the farmers perceived low behavioral control towards improving feeding.

### TPB factors

Towards all MCMs, the majority of the dairy farmers had a positive AT. The AT questions for nsMCMs were referring to the impact of mastitis, but AT questions of the specific MCMs were referring to the importance of those MCMs. This shows that dairy farmers recognize both the economic impact of mastitis and the importance of the specific MCMs. The percentage of farmers that had a positive SN was high except for nsMCMs. The reason may be due to the fact that nsMCMs are not a tangible measure, and therefore farmers may not have a clear idea to enact normative beliefs of referents.

The PBC of dairy farmers was high for three of the specific MCMs, but low for nsMCMs and for improving feeding of cows. The PBC was evaluated using statements referring to the ability of the dairy farmer to implement the MCM, availability of time and money to cover costs of the control measure. On average, 70% of the dairy farmers reported they have the ability, time and money to implement specific MCMs, but for nsMCMs and for improving feeding of cows, this was only 56% and 53%, respectively. Costs are the most important factor limiting the PBC of improving feeding. Feeding is very expensive so it is perceived that the profits are not justified by the costs. About the reasons behind the low PBC of nsMCMs, we speculate that this is a reflection of the fact that it is not a tangible measure and dairy farmers may assume it being impractical. A comparable study by Gunn et al. [40] reported that cattle and sheep farmers, who had a specific disease problem, but who assumed that methods were impractical or time consuming, were unwilling to practice biosecurity measures. If a measure is easy to implement, farmers are more motivated to implement the measure [39].

### Associations between TPB factors and intentions

Dairy farmers who had a positive AT had higher odds of intention to implement nsMCMs. We mentioned that, for nsMCMs, AT was evaluated by questions referring to the impact of mastitis, such as treatment costs and milk production losses. This is in line with Valeeva et al. [26], who described that monetary factors affect dairy farmers’ motivation to mastitis management. Similarly, dairy farmers who had a positive AT had higher odds of intention to improve stall hygiene and to implement foremilk stripping. Attitudes for the intentions to these specific MCMs were evaluated by questions referring to the benefits the farmer expected to have if he would implement these measures. Farmers who have evidence of effectiveness of a measure were motivated to implement those measures [39]. From the positive association between AT and intentions towards several MCMs, we conclude that information directed at improving farmers' understanding of the impact of mastitis and of the effect of interventions will likely increase practicing of MCMs.

Dairy farmers who had positive SN had higher odds of a positive intention towards udder cleaning and to implement foremilk stripping (Table 5). Many of the farmers were unfamiliar with the concept of foremilk stripping, those farmers may not have sufficient knowledge to develop their own beliefs and may therefore only have positive intention if the referents approve that foremilk stripping is important to improve udder health. Less experienced farmers, are more likely to be proactive in looking for up-to-date information on livestock disease [39]. In a comparable study on the operators’ non-compliance behavior to conduct emergency operating procedures, Park and Jung [41] found that relatively less experienced senior reactor operators’ who did not have sufficient knowledge to build up their own beliefs tend to rely on training they received and showed higher compliance. A reason for the association between SN and intention to perform udder cleaning may be due to the fact that in North-Western Ethiopian dairy farmers clean the udder not only for mastitis control but also to attract milk customers. Therefore, dairy farmers respond positively to the statement about their intention to clean udders and also have a strong positive SN. The way the milk is sold may therefore be a confounder in this association, but we have no data to support this. In this regard, veterinarians probably had the greatest influence on intention of dairy farmers towards cleaning of the udder.

The PBC of dairy farmers was high for udder cleaning, stall hygiene and foremilk stripping, but it was not significantly associated with any of the intentions. This shows that, when trying to improve mastitis control behavior, improving the PBC of the farmers may have less effect than, for instance, improving AT or SN. Still, the PBC is also a proxy for actual control, which has a direct link to behavior [42]. The PBC is always of importance, but as it was generally high, it is likely not the limiting factor in the Ethiopian situation. Therefore, interventions to improve udder health should be targeted at AT and SN rather than at PBC. Farm specific costs of mastitis should be calculated to show dairy farmers the extent mastitis costs them. Training and promotion on the advantages of mastitis control measures to dairy farmers themselves, to milk customers and neighbors as well as informing veterinarians and artificial inseminators about their influence on changing dairy farmers intention towards mastitis control are important. In addition, farmers often identify ‘other farmers’ as a major source of new ideas to uptake new technology [39]. Furthermore, farmers are strongly influenced by the perception whether a given measure is actually applicable on the farm [43]. Therefore, forming farmer groups as an opportunity for exchanging ideas and accessing advice and practical demonstration of the control measures may also be important to improve udder health in North-Western Ethiopia.

### Socio-demographic characteristics associated with TPB factors

Dairy farmers who experienced mastitis in their own farm in the previous year had more often a positive AT to implement nsMCMs compared to dairy farmers who did not experience this, indicating that the farmers’ behavior is influenced by experience of the disease, as was also reported by Garforth et al. [39]. Dairy farmers who had >15 years dairy farming experience had higher odds of a positive AT to implement foremilk stripping compared to less experienced dairy farmers. Since foremilk stripping is propagated by governmental extension workers, more experienced dairy farmers have a higher chance to have learned that foremilk stripping leads to earlier detection of mastitis enabling the farmer to take subsequent action. This may illustrate the effect of communication programs, as was also shown in the Netherlands, where planned communication about MCMs was shown to affect the ATs of farmers towards these measures [44]. Dairy farmers who had >8 grade level of education had lower odds of SN with regard to foremilk stripping. It is likely that farmers who have had more education feel more confident to implement mastitis control by themselves and be less susceptible to the advice of referents. Male farmers had lower SN to improve udder cleaning than female farmers. Our finding supports the work by Mekonnen et al. [10] who showed that women-owned farms had better animal care than men-owned farms.

We tested for associations between 9 background factors and the 3 TPB factors (AT, SN and PBC) for each of the 5 MCMs. The large number of comparisons (135) made in this way, results in a high chance of making type I errors. Still, the actual number of significant associations was only four, suggesting that social and demographic factors were hardly linked to the TPB factors. We discussed the factors that showed statistically significant associations, but for all of these, we should mention the fact that they may well have resulted from chance and have no biological interpretation.

## Conclusions

Our study identifies the determinants of dairy farmers’ motivation towards controlling mastitis. The majority of dairy farmers has a positive intention, and has a positive AT towards MCMs. However, SN and PBC for nsMCMs, PBC for feeding and SN for stall hygiene were low. Attitude and SN were positively associated with intentions towards several specific MCMs. Therefore, to increase the intention of dairy farmers to implement MCMs interventions directed at changing their AT and their SN are expected to give the largest improvement in mastitis control behavior in North-Western Ethiopian dairy farmers.

## Supporting information

S1 Table (DOCX)

S2 Table (CSV)
