# Supplementary material for: The intention of North-Western Ethiopian dairy farmers to control mastitis
Source: PLoS One. 2017 Aug 7;12(8):e0182727. doi: 10.1371/journal.pone.0182727 (PMC5546620; doi:10.1371/journal.pone.0182727)
Supplement: S1 Table — (DOCX) [file pone.0182727.s001.docx]

**Questionnaire on attitudes, subjective norms, perceived behavioral control and intentions of dairy farmers to control mastitis**

**I. Intention, attitude, subjective norms and perceived behavioural control to implement non-specified mastitis control measures**

**Intention: Implementation of non-specified mastitis control measures**

| **Intention** |  |
| --- | --- |
| In the near future I will implement one or more measures to reduce mastitis in my herd | Strongly disagree -3 -2 -1 0 1 2 3 Strongly agree |
| **Attitude** |  |
| **Behavioural beliefs** |  |
| Mastitis is expensive; therefore mastitis control is important | Strongly disagree 1 2 3 4 5 6 7 Strongly agree |
| Mastitis causes milk production losses, therefore mastitis control is important | Strongly disagree 1 2 3 4 5 6 7 Strongly agree |
| Mastitis is a risk of culling, therefore mastitis control is important | Strongly disagree 1 2 3 4 5 6 7 Strongly agree |
| Mastitis milk cannot be sold, therefore mastitis control is important | Strongly disagree 1 2 3 4 5 6 7 Strongly agree |
| **Outcome evaluation** |  |
| What is the importance of the following facts on your intention regarding preventive mastitis management |  |
| Increased costs for treatment of mastitis (more expensive drugs or veterinarians) | Very unimportant -3 -2 -1 0 1 2 3 Very important |
| Increased milk production losses due to mastitis | Very unimportant -3 -2 -1 0 1 2 3 Very important |
| Increased risk of culling due to mastitis | Very unimportant -3 -2 -1 0 1 2 3 Very important |
| Unable to sell mastitis milk | Very unimportant -3 -2 -1 0 1 2 3 Very important |
| **Subjective norm** |  |
| **Strength of normative belief** |  |
| What, according to your knowledge, is the opinion of the following people regarding the control of mastitis |  |
| Veterinarians think that control of mastitis is | Very unimportant -3 -2 -1 0 1 2 3 Very important |
| The artificial inseminator thinks that control of mastitis is | Very unimportant -3 -2 -1 0 1 2 3 Very important |
| My milk customers think that control of mastitis is | Very unimportant -3 -2 -1 0 1 2 3 Very important |
| My neighbours think that control of mastitis is | Very unimportant -3 -2 -1 0 1 2 3 Very important |
| Other dairy farmers think that control of mastitis is | Very unimportant -3 -2 -1 0 1 2 3 Very important |
| **Motivation to comply** |  |
| Does the opinion of the following people regarding reduction of mastitis influence your intention to implement preventive measures? |  |
| Veterinarians | Not at all 1 2 3 4 5 6 7 Very much |
| The artificial inseminator | Not at all 1 2 3 4 5 6 7 Very much |
| My milk customers | Not at all 1 2 3 4 5 6 7 Very much |
| My neighbours | Not at all 1 2 3 4 5 6 7 Very much |
| Other dairy farmers | Not at all 1 2 3 4 5 6 7 Very much |
| **Perceived behavioural control** |  |
| **Strength of control beliefs** |  |
| Management measures to reduce mastitis will be effective on my farm | Strongly disagree -3 -2 -1 0 1 2 3 Strongly agree |
| Management measures to reduce mastitis are expensive | Strongly disagree -3 -2 -1 0 1 2 3 Strongly agree |
| Management measures to reduce mastitis are time consuming | Strongly disagree -3 -2 -1 0 1 2 3 Strongly agree |
| Management measures to reduce mastitis are difficult to carry out | Strongly disagree -3 -2 -1 0 1 2 3 Strongly agree |
| **Power to influence behaviour** |  |
| I have money available to implement preventive management measures to reduce mastitis in my farm | Strongly disagree 1 2 3 4 5 6 7 Strongly agree |
| I have time to implement preventive management measures to reduce mastitis in my farm | Strongly disagree 1 2 3 4 5 6 7 Strongly agree |
| I have the ability/skill to implement preventive management measures to reduce mastitis in my farm | Strongly disagree 1 2 3 4 5 6 7 Strongly agree |

**II. Intensions, attitudes subjective norms and perceived behavioural control to implement specific mastitis control measures**

**Intention: Udder cleaning**

| In the near future I plan to improve the cleaning of the udders to reduce mastitis in my farm | Strongly disagree -3 -2 -1 0 1 2 3 Strongly agree |
| --- | --- |

**Attitude for udder cleaning**

| **Behavioural beliefs** |  |
| --- | --- |
| Udder cleaning improves milking hygiene | Strongly disagree 1 2 3 4 5 6 7 Strongly agree |
| Cleaning the udder minimizes spread of bugs causing mastitis | Strongly disagree 1 2 3 4 5 6 7 Strongly agree |
| Clean udders facilitate detection of mastitis | Strongly disagree 1 2 3 4 5 6 7 Strongly agree |
| **Outcome evaluation** |  |
| Milking hygiene by cleaning the udder is important to reduce mastitis | Strongly disagree -3 -2 -1 0 1 2 3 Strongly agree |
| Minimizing spread of bugs causing mastitis by cleaning the udder is important to reduce mastitis | Strongly disagree -3 -2 -1 0 1 2 3 Strongly agree |
| Easy detection of mastitis is important to reduce mastitis | Strongly disagree -3 -2 -1 0 1 2 3 Strongly agree |

**Subjective Norm for udder cleaning**

| What, according to your knowledge, is the opinion of the following people regarding the control of mastitis |  |
| --- | --- |
| Veterinarians think that udder cleaning is | Very unimportant 1 2 3 4 5 6 7 Very important |
| The artificial inseminator thinks that udder cleaning is | Very unimportant 1 2 3 4 5 6 7 Very important |
| My milk customers think that udder cleaning is | Very unimportant 1 2 3 4 5 6 7 Very important |
| My neighbours think that udder cleaning is | Very unimportant 1 2 3 4 5 6 7 Very important |
| Other dairy farmers think that udder cleaning is | Very unimportant 1 2 3 4 5 6 7 Very important |
| **Motivation to comply** |  |
| Does the opinion of the following people regarding udder cleaning influence your intention to improve udder cleaning? |  |
| Veterinarians | Not at all -3 -2 -1 0 1 2 3 Very much |
| The artificial inseminator | Not at all -3 -2 -1 0 1 2 3 Very much |
| My milk customers | Not at all -3 -2 -1 0 1 2 3 Very much |
| My neighbours | Not at all -3 -2 -1 0 1 2 3 Very much |
| Other dairy farmers | Not at all -3 -2 -1 0 1 2 3 Very much |

**Perceived Behavioural Control for udder cleaning**

| **Strength of Control Beliefs** |  |
| --- | --- |
| Udder cleaning will be effective on my farm to reduce mastitis | Strongly disagree 1 2 3 4 5 6 7 Strongly agree |
| Udder cleaning is difficult | Strongly disagree 1 2 3 4 5 6 7 Strongly agree |
| Cleaning the udder is time consuming | Strongly disagree 1 2 3 4 5 6 7 Strongly agree |
| Cleaning the udder is expensive | Strongly disagree 1 2 3 4 5 6 7 Strongly agree |
| **Power of Factors to Influence the Behaviour** |  |
| I know how to clean the udder | Strongly disagree -3 -2 -1 0 1 2 3 Strongly agree |
| I have time to clean the udder | Strongly disagree -3 -2 -1 0 1 2 3 Strongly agree |
| I can afford to cover costs of cleaning the udder | Strongly disagree -3 -2 -1 0 1 2 3 Strongly agree |

**Intention: Hand Cleaning**

| In the near future I plan to improve cleaning my hands while milking to reduce mastitis in my farm | Strongly disagree -3 -2 -1 0 1 2 3 Strongly agree |
| --- | --- |

**Attitude for hand cleaning**

| **Behavioural beliefs** |  |
| --- | --- |
| Milking with clean hands improves milking hygiene | Strongly disagree 1 2 3 4 5 6 7 Strongly agree |
| Milking with clean hands minimize spread of bugs causing mastitis among cows | Strongly disagree 1 2 3 4 5 6 7 Strongly agree |
| **Outcome evaluation** |  |
| Milking hygiene by cleaning hands is important to reduce mastitis | Strongly disagree -3 -2 -1 0 1 2 3 Strongly agree |
| Minimizing spread of mastitis bugs by cleaning hands is important to reduce mastitis | Strongly disagree -3 -2 -1 0 1 2 3 Strongly agree |

**Subjective Norm for hand cleaning**

| **Strength of normative beliefs** |  |
| --- | --- |
| What, according to your knowledge, is the opinion of the following people regarding the control of mastitis |  |
| Veterinarians think that hand cleaning is | Very unimportant -3 -2 -1 0 1 2 3 Very important |
| The artificial inseminator thinks that hand cleaning is | Very unimportant -3 -2 -1 0 1 2 3 Very important |
|  |  |
| My milk customers think that hand cleaning is | Very unimportant -3 -2 -1 0 1 2 3 Very important |
| My neighbours think that hand cleaning is | Very unimportant -3 -2 -1 0 1 2 3 Very important |
| Other dairy farmers think that hand cleaning is | Very unimportant -3 -2 -1 0 1 2 3 Very important |
| **Motivation to comply** |  |
| Does the opinion of the following people regarding hand cleaning influence your intention to improve hand cleaning? |  |
| Veterinarians | Not at all 1 2 3 4 5 6 7 Very much |
| The artificial inseminator | Not at all 1 2 3 4 5 6 7 Very much |
| My milk customers | Not at all 1 2 3 4 5 6 7 Very much |
| My neighbours | Not at all 1 2 3 4 5 6 7 Very much |
| Other dairy farmers | Not at all 1 2 3 4 5 6 7 Very much |

**Perceived Behavioural Control for hand cleaning**

| **Strength of Control Beliefs** |  |
| --- | --- |
| Cleaning hands will be effective on my farm to reduce mastitis | Strongly disagree 1 2 3 4 5 6 7 Strongly agree |
| Hand cleaning is difficult | Strongly disagree 1 2 3 4 5 6 7 Strongly agree |
| Cleaning the hands is time consuming | Strongly disagree 1 2 3 4 5 6 7 Strongly agree |
| Cleaning the hands is expensive | Strongly disagree 1 2 3 4 5 6 7 Strongly agree |
| **Power of Factors to Influence the Behaviour** |  |
| I know how to clean the hands | Strongly disagree -3 -2 -1 0 1 2 3 Strongly agree |
| I have time to clean the hands | Strongly disagree -3 -2 -1 0 1 2 3 Strongly agree |
| I can afford to cover costs of cleaning the hands | Strongly disagree -3 -2 -1 0 1 2 3 Strongly agree |

**Intention: Improving stall hygiene**

| In the near future, I plan to improve stall hygiene to reduce mastitis in my farm | Strongly disagree -3 -2 -1 0 1 2 3 Strongly agree |
| --- | --- |

**Attitude for stall hygiene**

| **Behavioural beliefs** |  |
| --- | --- |
| A clean stall reduces mastitis | Strongly disagree 1 2 3 4 5 6 7 Strongly agree |
| Cleaning the stall minimizes transmission of mastitis agents | Strongly disagree 1 2 3 4 5 6 7 Strongly agree |
| Improving the stall hygiene reduce exposure of teats to mastitis pathogens | Strongly disagree 1 2 3 4 5 6 7 Strongly agree |
| **Outcome evaluation** |  |
| Stall hygiene is important to reduce mastitis | Strongly disagree -3 -2 -1 0 1 2 3 Strongly agree |
| Minimizing transmission of mastitis agents by cleaning the stall is important to reduce mastitis | Strongly disagree -3 -2 -1 0 1 2 3 Strongly agree |
| Minimizing exposure of teats to mastitis pathogens is important to reduce mastitis | Strongly disagree -3 -2 -1 0 1 2 3 Strongly agree |

**Subjective Norm for stall hygiene**

| **Strength of normative beliefs** |  |
| --- | --- |
| What, according to your knowledge, is the opinion of the following people regarding the control of mastitis? |  |
| Veterinarians think that stall hygiene is | Very unimportant -3 -2 -1 0 1 2 3 Very important |
| The artificial inseminator thinks that stall hygiene is | Very unimportant -3 -2 -1 0 1 2 3 Very important |
| My milk customers think that stall hygiene is | Very unimportant -3 -2 -1 0 1 2 3 Very important |
| My neighbours think that stall hygiene is | Very unimportant -3 -2 -1 0 1 2 3 Very important |
| Other dairy farmers think that stall hygiene is | Very unimportant -3 -2 -1 0 1 2 3 Very important |
| **Motivation to comply** |  |
| Does the opinion of the following people regarding stall hygiene influence your intention to improve stall hygiene? |  |
| Veterinarians | Not at all 1 2 3 4 5 6 7 Very much |
| The artificial inseminator | Not at all 1 2 3 4 5 6 7 Very much |
| My milk customers | Not at all 1 2 3 4 5 6 7 Very much |
| My neighbours | Not at all 1 2 3 4 5 6 7 Very much |
| Other dairy farmers | Not at all 1 2 3 4 5 6 7 Very much |

**Perceived Behavioural Control for stall hygiene**

| **Strength of Control Beliefs** |  |
| --- | --- |
| An optimal stall hygiene will be effective on my farm to reduce mastitis | Strongly disagree 1 2 3 4 5 6 7 Strongly agree |
| Cleaning the stall is difficult | Strongly disagree 1 2 3 4 5 6 7 Strongly agree |
| Cleaning the stall is time consuming | Strongly disagree 1 2 3 4 5 6 7 Strongly agree |
| Cleaning the stall is expensive | Strongly disagree 1 2 3 4 5 6 7 Strongly agree |
| **Power of Factors to Influence the Behaviour** |  |
| I know how to clean the stall | Strongly disagree -3 -2 -1 0 1 2 3 Strongly agree |
| I have time to clean the stall | Strongly disagree -3 -2 -1 0 1 2 3 Strongly agree |
| I can afford to cover costs of cleaning the stall | Strongly disagree -3 -2 -1 0 1 2 3 Strongly agree |

**Intention: Improve Feeding**

| In the near future I plan to improve feeding of my cows to reduce mastitis in my farm | Strongly disagree -3 -2 -1 0 1 2 3 Strongly agree |
| --- | --- |

**Attitude for feeding Improvement**

| **Behavioural beliefs** |  |
| --- | --- |
| Appropriate feeding improves nutritional balance | Strongly disagree 1 2 3 4 5 6 7 Strongly agree |
| Appropriate feeding improves resistance to disease; hence to mastitis | Strongly disagree 1 2 3 4 5 6 7 Strongly agree |
| **Outcome evaluation** |  |
| Balanced feeding is important to reduce mastitis | Strongly disagree -3 -2 -1 0 1 2 3 Strongly agree |
| Improving resistance to mastitis is important to reduce mastitis | Strongly disagree -3 -2 -1 0 1 2 3 Strongly agree |

**Subjective Norm for feeding Improvement**

| What, according to your knowledge, is the opinion of the following people regarding the control of mastitis |  |
| --- | --- |
| Veterinarians think that improving feeding is | Very unimportant 1 2 3 4 5 6 7 Very important |
| The artificial inseminator thinks that improving feeding is | Very unimportant 1 2 3 4 5 6 7 Very important |
| My milk customers think that improving feeding is | Very unimportant 1 2 3 4 5 6 7 Very important |
| My neighbours that improving feeding is | Very unimportant 1 2 3 4 5 6 7 Very important |
| Other dairy farmers think improving feeding is | Very unimportant 1 2 3 4 5 6 7 Very important |
| **Motivation to comply** |  |
| Does the opinion of the following people regarding feeding improvement influence your intention to improve feeding? |  |
| Veterinarians | Not at all -3 -2 -1 0 1 2 3 Very much |
| The artificial inseminator | Not at all -3 -2 -1 0 1 2 3 Very much |
| My milk customers | Not at all -3 -2 -1 0 1 2 3 Very much |
| My neighbours | Not at all -3 -2 -1 0 1 2 3 Very much |
| Other dairy farmers | Not at all -3 -2 -1 0 1 2 3 Very much |

**Perceived Behavioural Control for feeding Improvement**

| **Strength of Control Beliefs** |  |
| --- | --- |
| Improvement in feeding will be effective on my farm to reduce mastitis | Strongly disagree 1 2 3 4 5 6 7 Strongly agree |
| Improvement in feeding is difficult | Strongly disagree 1 2 3 4 5 6 7 Strongly agree |
| Improvement in feeding is time consuming | Strongly disagree 1 2 3 4 5 6 7 Strongly agree |
| Improvement in feeding is expensive | Strongly disagree 1 2 3 4 5 6 7 Strongly agree |
| **Power of Factors to Influence the Behaviour** |  |
| I know how to improve feeding | Strongly disagree -3 -2 -1 0 1 2 3 Strongly agree |
| I have time to improve feeding | Strongly disagree -3 -2 -1 0 1 2 3 Strongly agree |
| I can afford to cover costs to improve feeding | Strongly disagree -3 -2 -1 0 1 2 3 Strongly agree |

**Intention: Foremilk stripping**

| In the near future I plan to foremilk strip to reduce mastitis in my farm | Strongly disagree -3 -2 -1 0 1 2 3 Strongly agree |
| --- | --- |

**Attitude foremilk stripping**

| **Behavioural beliefs** |  |
| --- | --- |
| Foremilk stripping is important to diagnose mastitis | Strongly disagree 1 2 3 4 5 6 7 Strongly agree |
| Foremilk stripping is important to decide treatment against mastitis | Strongly disagree 1 2 3 4 5 6 7 Strongly agree |
| **Outcome evaluation** |  |
| Diagnosis of mastitis by foremilk stripping is important to reduce mastitis | Strongly disagree -3 -2 -1 0 1 2 3 Strongly agree |
| Treatment decision against mastitis by foremilk stripping is important to reduce mastitis | Strongly disagree -3 -2 -1 0 1 2 3 Strongly agree |

**Subjective Norm for foremilk stripping**

| **Strength of normative beliefs** |  |
| --- | --- |
| What, according to your knowledge, is the opinion of the following people regarding foremilk stripping to control mastitis |  |
| Veterinarians think that foremilk stripping is | Very unimportant -3 -2 -1 0 1 2 3 Very important |
| The artificial inseminator thinks that foremilk stripping is | Very unimportant -3 -2 -1 0 1 2 3 Very important |
| My milk customers think that foremilk stripping is | Very unimportant -3 -2 -1 0 1 2 3 Very important |
| My neighbours think that foremilk stripping is | Very unimportant -3 -2 -1 0 1 2 3 Very important |
| Other dairy farmers think that foremilk stripping is | Very unimportant -3 -2 -1 0 1 2 3 Very important |
| **Motivation to comply** |  |
| Does the opinion of the following people regarding foremilk stripping influence your intention to control mastitis? |  |
| Veterinarians | Not at all 1 2 3 4 5 6 7 Very much |
| The artificial inseminator think | Not at all 1 2 3 4 5 6 7 Very much |
| My milk customers | Not at all 1 2 3 4 5 6 7 Very much |
| My neighbours | Not at all 1 2 3 4 5 6 7 Very much |
| Other dairy farmers | Not at all 1 2 3 4 5 6 7 Very much |

**Perceived Behavioural Control for foremilk stripping**

| **Strength of Control Beliefs** |  |
| --- | --- |
| Foremilk stripping will be effective on my farm to reduce mastitis | Strongly disagree 1 2 3 4 5 6 7 Strongly agree |
| Foremilk stripping is difficult | Strongly disagree 1 2 3 4 5 6 7 Strongly agree |
| Foremilk stripping is time consuming | Strongly disagree 1 2 3 4 5 6 7 Strongly agree |
| Foremilk stripping is expensive | Strongly disagree 1 2 3 4 5 6 7 Strongly agree |
| **Power of Factors to Influence the Behaviour** |  |
| I know how to make foremilk stripping | Strongly disagree -3 -2 -1 0 1 2 3 Strongly agree |
| I have time to make foremilk stripping | Strongly disagree -3 -2 -1 0 1 2 3 Strongly agree |
| I can afford to cover costs of to make foremilk stripping | Strongly disagree -3 -2 -1 0 1 2 3 Strongly agree |
